# Epimorphic development in tropical shallow-water Nymphonidae (Arthropoda: Pycnogonida) revealed by fluorescence imaging

**DOI:** 10.1186/s40851-023-00223-8

**Published:** 2024-01-02

**Authors:** Claudia P. Arango, Georg Brenneis

**Affiliations:** 1 Queensland Museum, Biodiversity Program, PO Box 3300, South Brisbane, QLD 4101 Australia; 2 Department Evolutionary Biology, Unit Integrative Zoology, University of Vienna, Djerassiplatz 1, 1030 Vienna, Austria

**Keywords:** Sea spiders, Pantopoda, Evolution, Development, Larva, Thorson’s rule, Scanning electron microscopy, Autofluorescence imaging, Confocal laser-scanning microscopy

## Abstract

**Background:**

Extant lineages of sea spiders (Pycnogonida) exhibit different types of development. Most commonly, pycnogonids hatch as a minute, feeding protonymphon larva with subsequent anamorphic development. However, especially in cold water habitats at higher latitudes and in the deep sea, some taxa have large, lecithotrophic larvae, or even undergo extended embryonic development with significantly advanced postlarval hatching stages. Similar biogeographic trends are observed in other marine invertebrates, often referred to as “Thorson’s rule”.

**Results:**

To expand our knowledge on the developmental diversity in the most speciose pycnogonid genus *Nymphon*, we studied the developmental stages of the two tropical representatives *N. floridanum* and *N. micronesicum*., We compared classical scanning electron microscopy with fluorescence-based approaches to determine which imaging strategy is better suited for the ethanol-fixed material available. Both species show epimorphic development and hatch as an advanced, lecithotrophic postlarval instar possessing the anlagen of all body segments. Leg pairs 1–3 show a considerable degree of differentiation at hatching, but their proximal regions remain coiled and hidden under the cuticle of the hatching instar. The adult palp and oviger are not anteceded by three-articled larval limbs, but differentiate directly from non-articulated limb buds during postembryonic development.

**Conclusions:**

Fluorescence imaging yielded more reliable morphological data than classical scanning electron microscopy, being the method of choice for maximal information gain from rare and fragile sea spider samples fixed in high-percentage ethanol. The discovery of epimorphic development with lecithotrophic postlarval instars in two small *Nymphon* species from tropical shallow-water habitats challenges the notion that this developmental pathway represents an exclusive cold-water adaptation in Nymphonidae. Instead, close phylogenetic affinities to the likewise more direct-developing Callipallenidae hint at a common evolutionary origin of this trait in the clade Nymphonoidea (Callipallenidae + Nymphonidae). The lack of functional palpal and ovigeral larval limbs in callipallenids and postlarval hatchers among nymphonids may be a derived character of Nymphonoidea. To further test this hypothesis, a stable and well-resolved phylogenetic backbone for Nymphonoidea is key.

## Background

Pycnogonida (sea spiders) is an old lineage of the Chelicerata (spiders, scorpions, horseshoe crabs, and relatives) that diverged from the remaining chelicerate taxa in the Paleozoic, potentially already in the Cambrian [1–3]. They contribute to marine benthic communities at virtually all latitudes and water depths in the world’s oceans [4]. The majority of extant species possess a comparatively narrow body that is equipped with an anterior proboscis, and typically bears an ocular tubercle with two pairs of single-lensed median eyes [5, 6], four pairs of long legs receiving diverticula from the midgut and gonads [7–10], and a tiny posterior anal tubercle. Remarkably, sea spiders exhibit paternal brood care [11], a rare trait among arthropods and dioecious invertebrates in general [12]. During mating, the male glues the eggs into one or two packages and carries them attached to the ovigers, a modified limb pair found anterior to the first ambulatory leg [3, 4], until hatching.

The development of many pycnogonid taxa is poorly understood and, in the case of some families (Austrodecidae, Colossendeidae, Rhynchothoracidae), completely unknown [13, 14]. Over the last two decades, only a handful of developmental studies have addressed aspects of pycnogonid embryology [15–18]. By contrast, the postembryonic developmental phase has been investigated at broader taxonomic range [19–28], mostly limited to documentation of eidonomy via scanning electron microscopy (SEM). Internal anatomical details are scarcer and predominantly studied in shallow-water species that can be readily collected near marine biological stations and maintained, at least temporarily, in the laboratory [17, 29–36].

The most common type of pycnogonid development features a free-living protonymphon larva as hatching stage with only three anterior appendage pairs (cheliphore plus larval palpal and ovigeral limbs) [13]. After attaching to a suitable host to feed on, this larva undergoes anamorphic development characterized by a stereotypical series of molts and the sequential differentiation of the leg-bearing trunk segments [35, 37], representing developmental type 1 sensu [13]. Deviating from this likely plesiomorphic mode, some pycnogonids produce much larger, yolk-rich eggs that provide prolonged lecithotrophic nutrition for the offspring. In these cases, either (1) a large, non-feeding protonymphon-like larva hatches and stays attached to the paternal ovigers while fueling trunk differentiation and several molts with its yolk reserves [23, 24, 38] (= type 2 sensu [13]), or alternatively (2) extended embryonic development occurs, terminating in a postlarval hatching stage with a significantly advanced level of trunk differentiation [19, 27–29, 39] (= type 5 sensu [13]). While such prolonged rearing of lecithotrophic offspring occurs in different pycnogonid families, it remains almost exclusively limited to species inhabiting higher latitudes. For this reason, it is generally assumed to have evolved multiple times within Pycnogonida, as an adaption to low-temperature habitats [13, 37, 40]. This pattern is similar to biogeographic tendencies observed in other marine invertebrates, often referred to as “Thorson’s rule” (e.g. [41–44]).

When studying pycnogonids and the diversity of small benthic invertebrates (< 5 mm) in general, the challenges of collecting, sorting and optimally preserving specimens increase with the remoteness and inaccessibility of sampling localities, where on-site processing time is a limiting factor. As a consequence, more often than not large quantities of unsorted samples of benthic organisms are immediately preserved in ethanol to ensure high DNA quality for subsequent identification and analyses. However, for the delicate developmental stages of sea spiders, such harsh dehydration may cause significant artefactual distortion and collapse of weakly sclerotized structures, thus proving detrimental for morphological investigation.

In this study, we analyzed late embryonic stages and postembryonic instars of two nymphonid species, *Nymphon floridanum* Hedgpeth, 1948 and *N. micronesicum* Child, 1982, to provide first insight into the hitherto unknown development of tropical representatives of the genus *Nymphon*. With more than 250 currently accepted species, *Nymphon* forms the most speciose pycnogonid genus [45], accounting for almost a fifth of all sea spider species described. Its species richness is accompanied by a cosmopolitan distribution, with members occurring from intertidal habitats to the deep sea, and in tropical, temperate, and polar benthic communities (e.g. [46–54]). Moreover, together with the ammotheid genus *Ammothea*, *Nymphon* displays the highest intra-generic diversity of developmental pathways documented in Pycnogonida [13, 22], with different species falling into types 1, 2, or 5 [28, 34, 35, 38, 55–60]. Upon collection, ovigerous/larvigerous males of *N. floridanum* and *N. micronesicum* were directly placed in high-percentage ethanol, without any prior tissue preservation (e.g., in formaldehyde-based fixative). We subjected part of the developmental material to classical SEM documentation widely used in the field and in parallel performed fluorescent nuclear staining and/or autofluorescence microscopy with the remaining samples. This mixed strategy was adopted to evaluate which approach yields more reliable structural information and should therefore be given preference for morphological analysis of rare and fragile pycnogonid material preserved in ethanol.

## Material and methods

### Sample collection and species identification

Embryonic and postembryonic developmental stages studied were taken from ovigerous males that had been preserved in 70–90% ethanol upon collection. Individual specimens of *Nymphon floridanum* were found in epifaunal samples collected as part of larger studies of epibenthos in the Caribbean, and four males of *N. micronesicum* were found in an inshore reef of the Central section of the Great Barrier Reef (Table 1). Species determination for *N. micronesicum* is documented in [46]. *N. floridanum* was determined following various taxonomic works by Hedgpeth, Stock and Child (e.g. [48, 61, 62]). For detailed analysis of the developmental stages, these were stripped from the males’ ovigers with tweezers.

**Table 1:** Locations and sampling details of *Nymphon* specimens studied

Species	Family	Material studied	Year of collection	Sampling locality
*Nymphon floridanum* Hedgpeth, 1948	Nymphonidae	1 ovigerous male	2002	Coral rubble (20 m depth), outer ridge reef, Carrie Bow Cay, Belize (see [63])
*Nymphon micronesicum* Child, 1982	Nymphonidae	4 ovigerous males	2000	Coral rubble (4–6 m depth), Pandora Reef, Great Barrier Reef, Australia (see [46])

### Fluorescent nuclear staining, data acquisition and analysis

Samples were rehydrated via a descending ethanol series into double-distilled water (ddH_2_O). Nuclear staining with the fluorescent nucleic acid marker Sytox®Green (Invitrogen Molecular Probes®, 1:1000 in ddH_2_O) was performed overnight at 4 °C.

Z-stacks of autofluorescence and Sytox®Green-stained specimens were taken with a Zeiss Lumar V12 and automatically aligned with Zeiss AxioVision software (Version 4.7.10, RRID:SCR_002677). Each aligned z-stack was subsequently merged to a single image with extended depth of field using Helicon Focus software (ver. 7.6.6, Helicon Soft Ltd., Kharkiv, Ukraine, RRID:SCR_014462).

Additionally, some embryonic and postlarval specimens were mounted on microscopic slides either (1) in ddH_2_O or (2) after transfer into RapiClear® 1.49 (SUNJin Lab, # RC149001) for tissue clearing. Cuticular autofluorescence and/or nuclear staining of the samples were imaged with a Leica DM IRE2 confocal laser-scanning microscope (CLSM) equipped with a Leica TCS SP2 AOBS laser-scan unit (RRID:SCR_018714). CLSM data visualization was performed with the 3D reconstruction software Imaris (Bitplane AG, Switzerland, version 7.0.0, RRID:SCR_007370) as previously described, including the generation of 3D-curved optical sections by combination of several oblique slicers [64].

### Scanning electron microscopy (SEM)

Pre-hatching embryos and hatched instars of *N. floridanum* were dehydrated via a graded ethanol series (70%, 80%, 90%, 96%, 2 × 100%, each step at least 30 min), critical point-dried with a Bal-Tec CPD 030 and sputtered with gold using a Bal-Tec SCD 005. Micrographs were taken with a Zeiss LEO 1430 scanning electron microscope. In an attempt to counteract the cuticular collapse and folding introduced by sample fixation in ethanol, some of the rehydrated *N. floridanum* specimens documented by fluorescent imaging were again carefully dehydrated in an ascending ethanol series for subsequent SEM analysis.

### Applied terminology and data presentation

Pycnogonid species names have been updated to current suggestions in Pycnobase [45]. The distinction and designation of developmental stages as larva or postlarval instars follows the definitions suggested in [13]. Further, in line with the reasoning outlined in [65], the posterior-most region of the pycnogonid body is neutrally designated as “anal tubercle”, instead of “abdomen”, which is widely used in the taxonomic literature.

Body length measurement was performed on SEM micrographs and fluorescence images along a straight line between the anterior tip of the body (ocular tubercle, if present) to its posterior-most tip (anal tubercle, if present). Limb pairs and proboscis were excluded from body length measurements. Global contrast and brightness values of some of the images were adjusted using Adobe Photoshop (ver. 12.1, Adobe Systems Incorporated, San Jose, CA, USA, RRID:SCR_014199). All figures were compiled with Adobe Illustrator (ver. 15.1, Adobe Systems Incorporated, RRID:SCR_010279).

## Results

### *Nymphon floridanum* Hedgpeth, 1948

The material studied was obtained from one male (Table 1), bearing a total of 25–30 young specimens belonging to four different developmental stages on its ovigers.

#### Late embryonic morphogenesis (2 × SEM); Fig. 1

**Fig. 1:**
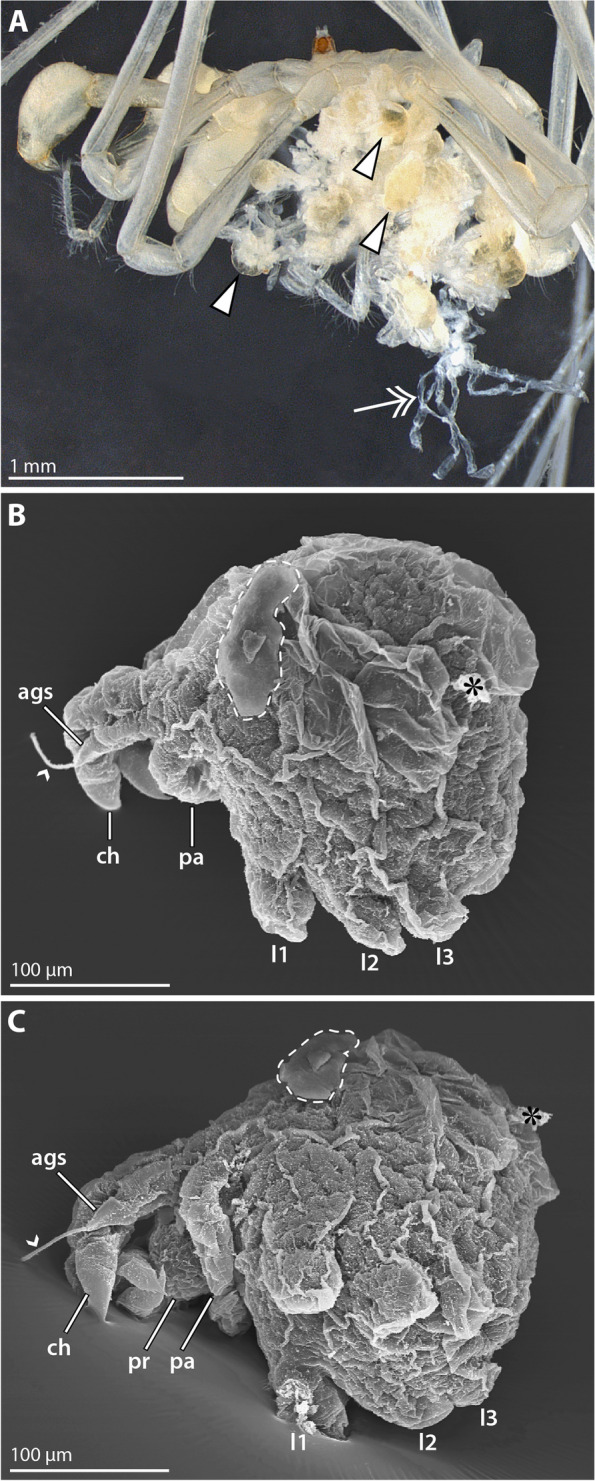
Ovigerous male and late embryo of *Nymphon floridanum*. **A** Adult male bearing different postembryonic instars; lateral view; stereomicroscopic image. At first, the attaching instars are equipped with yellowish yolk reserves (arrowheads). In postlarval instar 3 (double arrow) the yolk has been exhausted. Note significant collapse and folding of the ethanol-preserved instar 3 specimen. **B** and **C** Pre-hatching embryo with cheliphores and anlagen of palps and leg pairs 1–3; SEM micrographs showing lateral (**B**) and ventrolateral (**C**) views. The embryonic cuticle is extensively folded. The small arrowhead points to fibrous secretions that project from the attachment gland spine’s distal tip. The stippled outline marks a remaining piece of the manually removed egg membrane. The asterisk labels a dirt particle. Abbreviations: ags – attachment gland spine; ch – cheliphore; l1-3 – leg pairs 1–3; pa – palp; pr – proboscis

Two embryos in a late stage of embryonic morphogenesis were found among the otherwise completely hatched offspring (Fig. 1). The embryos measure approximately 250 µm along the anteroposterior axis. They possess an embryonic cuticle that shows considerable wrinkles and folds (Fig. 1B, C), partially attributable to the ethanol preservation of the collected material. The anteroventrally directed proboscis anlage is flanked by the chelate cheliphore bearing a well-developed attachment gland spine; fibrous secretions emanate from a pore at the spine’s tip (Fig. 1B, C). The cheliphore is posteriorly followed by the elongate, ventromedially extending bud of the prospective palp that shows no detectable signs of articulation. No oviger anlage is discernible. Short limb buds of leg pairs 1–3 protrude ventrally from the wrinkled cuticle (Fig. 1B, C), indicative of an advanced differentiation of trunk segments during embryonic development of *N. floridanum*.

#### Hatching stage = postlarval instar 1 (2 × Sytox; 2 × SEM); Fig. 2

**Fig. 2:**
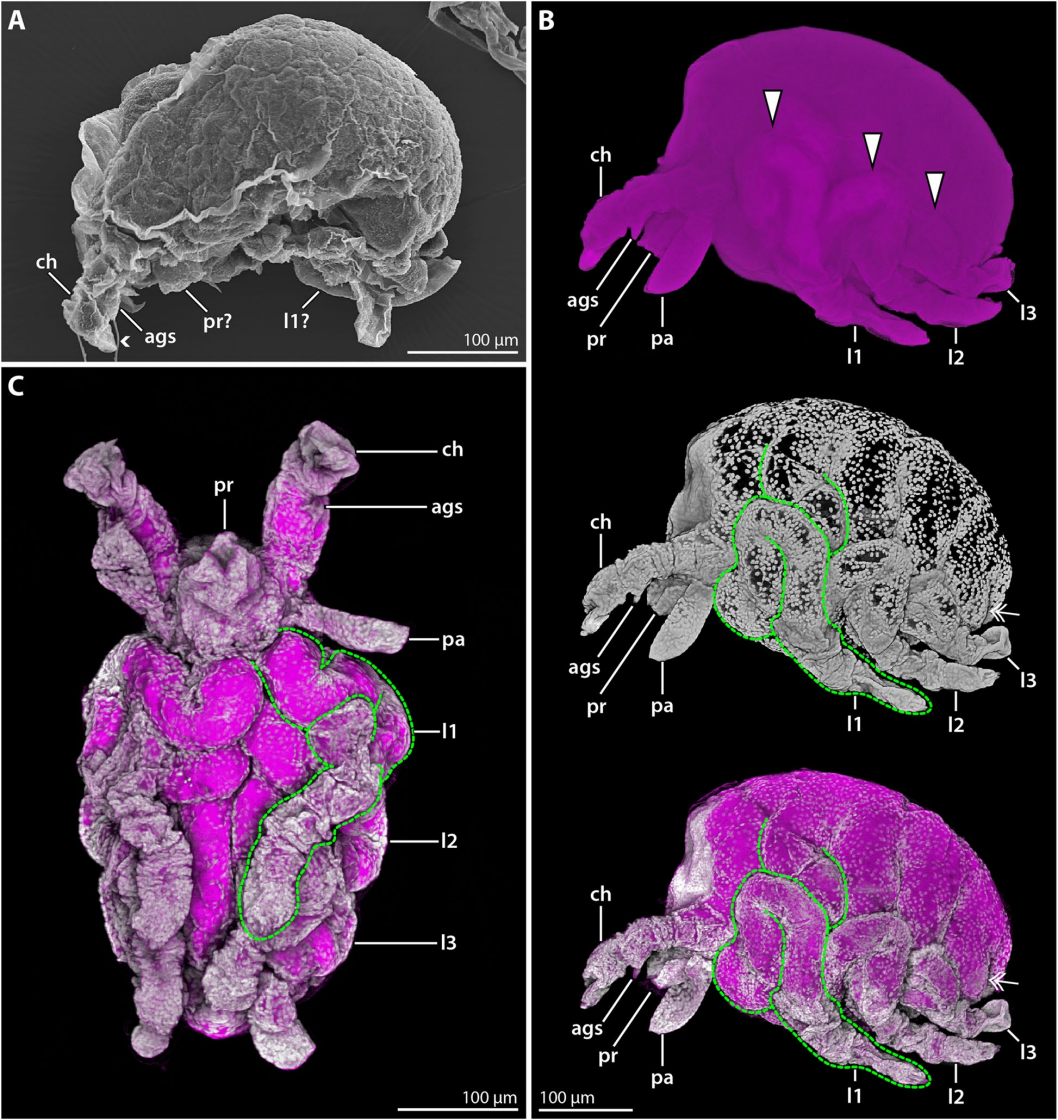
Hatching instar 1 of *Nymphon floridanum*. Comparison of SEM and CLSM imaging. **A** Lateral view; SEM. All appendages are collapsed and difficult to distinguish. The small arrowhead marks a fibrous strand emanating from the cheliphoral attachment gland spine. **B** Lateral view; CLSM; autofluorescence (magenta) and nuclear staining (gray) shown as single channels (blend mode) and combined (maximum intensity projection). The yolk-filled proximal portions of leg pairs 1–3 are coiled beneath the cuticle (arrowheads). The green stippled line exemplarily outlines leg 1. The double arrow marks the tiny bud of leg 4. **C** Ventral view; autofluorescence and nuclear staining (maximum intensity projection, same colors as in **B**). The folded leg pairs 1–3 cover the ventral side of the trunk. Abbreviations: ags – attachment gland spine; ch – cheliphore; l1-3 – leg pairs 1–3; pa – palp; pr – proboscis

The hatching instar measures about 350 µm along the anteroposterior axis. It is lecithotrophic and contains a copious amount of yellow-transparent yolk (Fig. 1A). In SEM-processed specimens, the appendages are significantly collapsed, rendering structural analysis virtually non-informative (Fig. 2A). Also in specimens subjected to rehydration and fluorescent nuclear staining, only very limited unfolding of the collapsed appendages was observed. In combination with the native autofluorescence of the samples, however, this staining revealed at least some more structural details.

The chelate cheliphore is protruding anteriorly and inserts anterolateral to the short proboscis (Fig. 2B, C). The attachment gland spine emanates ventrally from the cheliphore’s scape region (Fig. 2) and in some of the specimens studied, fibrous secretions were found to project from its tip (Fig. 2A). The cheliphore is posteriorly adjoined by the palp, which still resembles an elongate, non-articulated bud (Fig. 2B, C). An oviger anlage was not detected. Externally, leg pairs 1–3 appear to be relatively short limb buds covered by (collapsed) cuticle (Fig. 2B). By contrast, nuclear staining reveals that the legs are already considerably longer and that only their distalmost portions are contained in the externally protruding cuticular buds. Underneath the cuticle, substantial proximal leg portions are coiled in snake-like fashion along the proximodistal axis, covering the ventrolateral sides of the trunk (Fig. 2B, C). These externally hidden leg portions contain autofluorescent yolk, presumably located in developing midgut diverticula. A tiny bud of leg 4 flanks the posterior body pole, being likewise hidden beneath the cuticle (Fig. 2B).

#### Postlarval instar 2 (3 × autofluorescence, 2 × SEM); Fig. 3

**Fig. 3:**
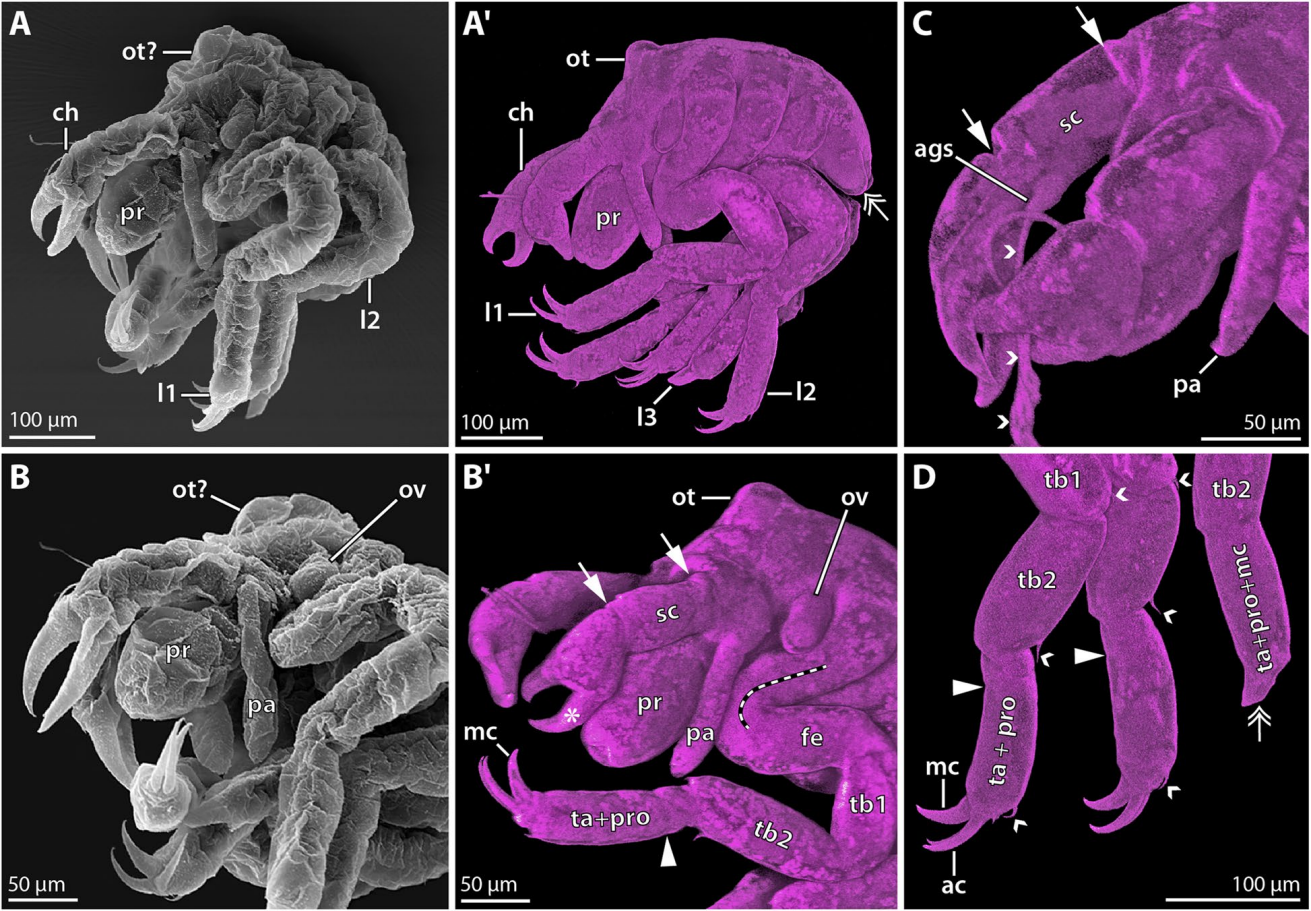
Instar 2 of *Nymphon floridanum*. Comparison of SEM and CLSM autofluorescence imaging. **A** Lateral view; SEM. The surface of the entire instar is significantly collapsed. **A’** Lateral view; CLSM. Gradual rehydration into distilled water has reversed the ethanol-induced structural collapse. The double arrow marks the small limb bud of leg 4. **B** Anterolateral view of the anterior body region; SEM. The cuticular collapse hampers reliable assessment of cheliphore structure, proboscis shape and the presence of the ocular tubercle. **B’** Anterolateral view of anterior body region; CLSM. The cheliphore’s scape borders (arrows) and moveable finger (asterisk) are recognizable. The ocular tubercle and barrel-shaped proboscis can be readily discerned. The arrowhead points to an indistinct division in the tarsus–propodus precursor. The stippled line indicates the leg region comprising coxae 1–3. **C** Detail of the cheliphores; CLSM. White arrows highlight the scape borders. Small arrowheads mark fibrous secretions emanating from the attachment gland spine. **D** Detail of the distal podomeres of legs 1–3; CLSM. Large arrowheads mark a notch near the proximal margin of the tarsus-propodus precursor of legs 1 and 2. Small arrowheads point to setae. The double arrow highlights the tip of leg 3 that still lacks the main and auxiliary claws. Abbreviations: ac – auxiliary claw; ags – attachment gland spine; ch – cheliphore; l1–3 – leg pairs 1–3; mc – main claw; pa – palp; ot – ocular tubercle; ov – oviger; pr – proboscis; sc – scape; ta + pro – tarsus–propodus precursor; ta + pro + mc – tarsus-propodus–main claw precursor; tb1 – tibia 1; tb2 – tibia 2

Also in postlarval instar 2, the cuticle was significantly collapsed and wrinkled in specimens studied with SEM (Fig. 1A; 3A, B). Contrary to the preceding instar, however, gradual rehydration into distilled water for fluorescence imaging resulted in an unfolding and smoothening of the cuticular surface (Fig. 3A′, B′, C, D). Careful re-dehydration of some unfolded specimens for another SEM experiment resulted in the re-collapse of the previously smooth cuticular surfaces, starting at approximately 60–70% ethanol.

The anteroposterior body extension of postlarval instar 2 lies in the same range as in instar 1, but the fully unfolded leg pairs 1–3 increase its overall volume markedly. Anterodorsally, a shallow, rounded ocular tubercle has formed (Fig. 3A’, B’). The anteroventrally directed proboscis is barrel-shaped (Fig. 3A′, B′). The cheliphore is three-articled, featuring a distinctly set off scape and the distal chela (Fig. 3B′, C). The chela overreaches the proboscis tip and possesses strong and slightly curved fingers without teeth that are approximately as long as the palm (Fig. 3A′-B′). The short attachment gland spine emerges medioventrally from the distal margin of the scape (Fig. 3C). The non-articulated palp bud is of similar length as the proboscis (Fig. 3A–B′). A small bulbous bud has emerged posterior to the palp, representing the oviger anlage (Fig. 3 A–B′). Leg pairs 1 and 2 distally bear the slightly curved main claw and paired auxiliary claws of the same length (Fig. 3A′, B′, D). In both leg pairs, the complete set of podomeres appears to be differentiated, but podomere borders are not clearly distinguishable proximally (coxae 1–3) and between the propodus and tarsus (Fig. 3B′, D). The longer podomeres (femur, tibiae 1 and 2, propodus) each bear at least one small dorsodistal seta (Fig. 3D). In leg pair 3, the main claw with its auxiliary claws, propodus and tarsus are hidden beneath an undivided cuticular cover and any setae are lacking (Fig. 3A’, D). Leg pair 4 remains a tiny bud that flanks the posterior body pole (Fig. 3A′).

#### Postlarval instar 3

A single specimen of postlarval instar 3 was still attached to one of the male’s ovigers (Fig. 1A). At this stage, the yolk reserves are nearly exhausted, suggesting that this instar is the one that abandons the father. Likely due to the absence of the dense yolk, the specimen was even more dramatically shrunken by the ethanol fixation than the specimens described above (Fig. 1A) and could not be restored by rehydration. Apart from the presence of an elongate leg pair 4, no additional structural details were documented.

### *Nymphon micronesicum* Child, 1982

Three different developmental stages (4 × embryonic morphogenesis; 8 × hatching instar; > 40 × instar 2) were obtained from four males preserved in 70% ethanol (Table 1). Owing to the suboptimal results obtained for *N. floridanum*, no further SEM processing was performed on the *N. micronesicum* material.

#### Embryonic morphogenesis (4 × Sytox); Fig. 4

**Fig. 4:**
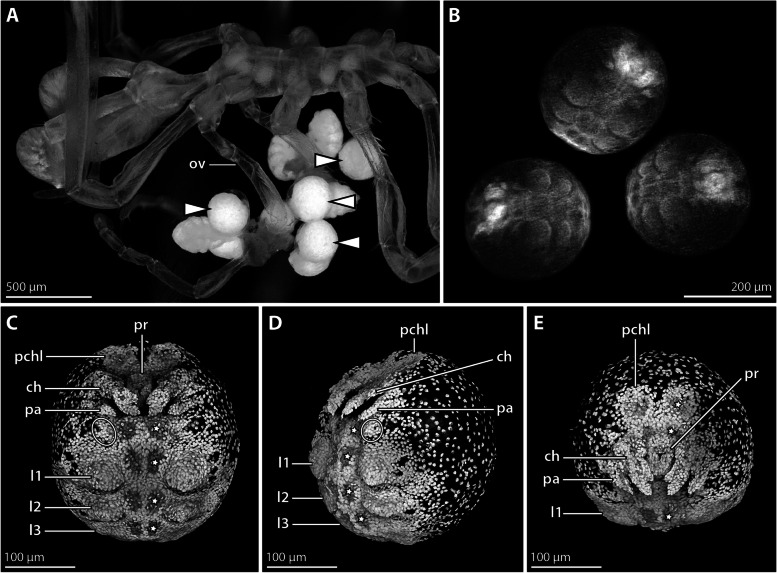
Advanced embryonic stage of *Nymphon micronesicum*. **A** Lateral view of adult male bearing embryos and hatched instars; stereomicroscopic autofluorescence image. White arrowheads indicate spherical embryos still contained in the egg membranes. **B** Embryos after Sytox staining; epifluorescence image. **C-E** CLSM scans of Sytox-stained embryos (blend mode). Stars mark one side of paired segmental depressions related to nervous system development (“ventral organs”). The ovals indicate the putative primordium of the future oviger. **C** Ventral view. **D** Ventrolateral view. **E** Anterior view. Abbreviations: ch – cheliphore; l1–3 – leg pairs 1–3; ov – oviger; pa – palp; pchl – pre-cheliphoral lobe; pr – proboscis

Embryos in advanced morphogenesis are of spherical shape, with slightly more than 200 µm diameter (Fig. 4A, B). They contain a considerable amount of yolk that shows strong autofluorescence under UV light (Fig. 4A). Nuclear staining reveals a condensed germ band that covers the ventral hemisphere of the embryo, whereas its dorsal hemisphere features only a thin layer of evenly spaced nuclei (Fig. 4B, D, E). The paired precheliphoral lobe forms the anterior-most region, posteriorly adjoined by the proboscis and cheliphore anlagen. The cheliphore inserts lateral to the proboscis and extends medioventrally around it (Fig. 4C, E). At its distal end, two lobes represent the primordia of the prospective chela fingers (Fig. 4C, E). Posterior to the cheliphore, a small limb bud of the prospective palp is discernible, followed by a minute cell condensation that may represent the primordium of the oviger (Fig. 4C–E). In the posterior half of the germ band, primordial limb buds of leg pairs 1–3 are developed. Central areas with internally displaced nuclei are found in the precheliphoral lobe, anterolateral to the proboscis and medial to the limb buds along the posterior germ band (Fig. 4C–E). They highlight the location of the so-called “ventral organs”, which are sites of embryonic and postembryonic neurogenesis (see [18]).

#### Hatching stage = postlarval instar 1 (8 × Sytox); Fig. 5

**Fig. 5:**
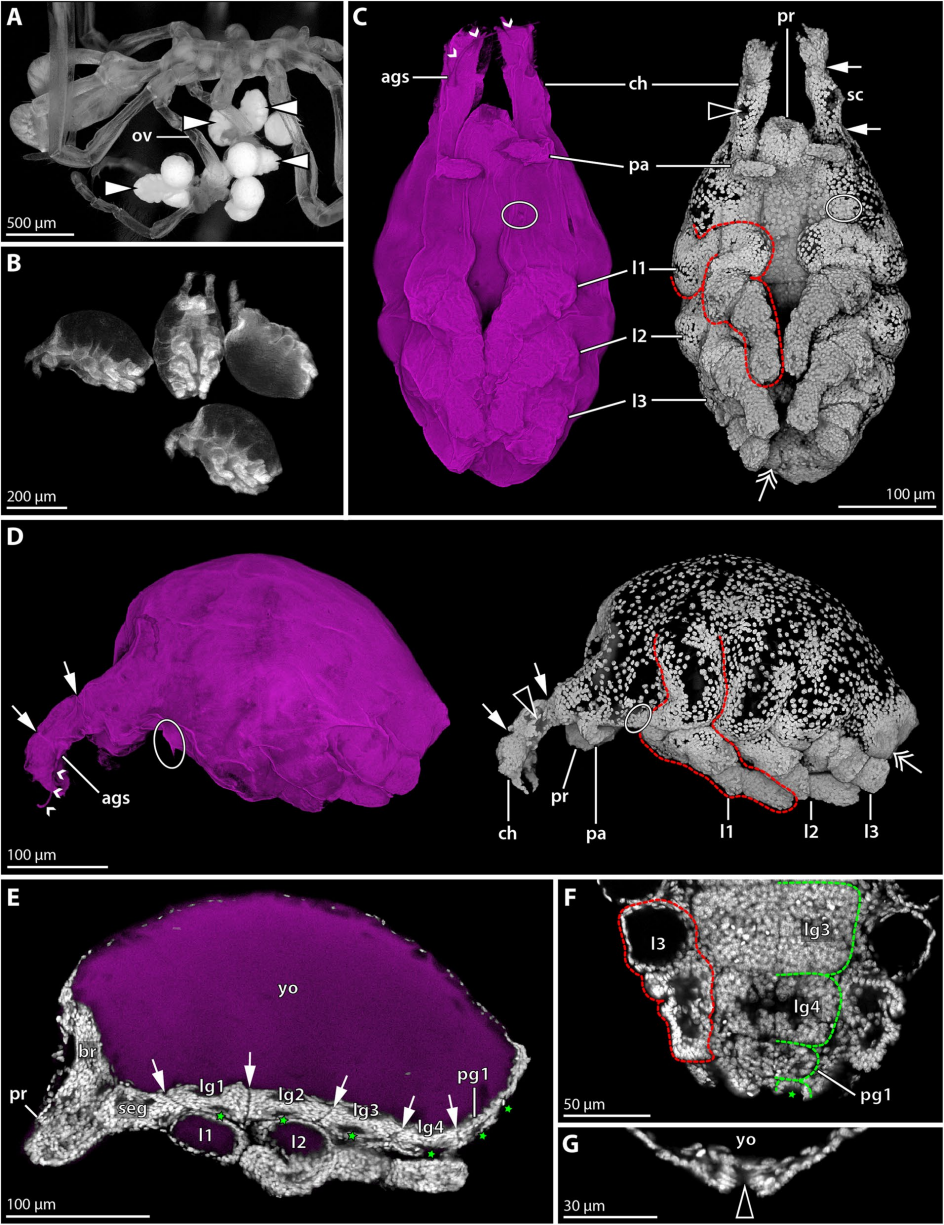
Hatching instar 1 of *Nymphon micronesicum*. **A** Lateral view of adult male bearing embryos and hatched instars; stereomicroscopic autofluorescence image. White arrowheads point to the ovoid-shaped hatching instar 1. **B** Instar 1 after Sytox staining, epifluorescence image. **C** and **D** Ventral and lateral views, respectively. CLSM scans (blend mode) of cuticular autofluorescence (magenta) and Sytox staining (gray) shown separately. White arrows indicate the scape boundaries. Small white arrowheads trace fibrous secretions projecting from the attachment gland spine. Black arrowheads point to the putative area of the attachment gland’s reservoir cells. The ovals mark the incipient oviger bud. The red stippled outline exemplarily highlights leg 1 that is folded and partially hidden under the cuticular cover. The double arrow points to the small bud of leg 4. **E** Virtual parasagittal section (Imaris oblique slicers), CLSM scan of autofluorescence (magenta) and Sytox staining (gray). The arrows mark borders between ventral ganglia. The green stars lie next to ventral organ cavities/invaginations. **F**&**G** Virtual horizontal sections (Imaris oblique slicers) of the posterior body pole, CLSM scans of Sytox staining. **F** The red outline marks leg 3 of one body half. The green stippled lines highlight the ganglion anlagen in the other body half. The primordial invagination of the ultimate ventral organ (green star) is visible posteriorly. **G** The proctodeum (arrowhead) forms at the tip of the anal tubercle. Abbreviations: ags – attachment gland spine; br – brain; ch – cheliphore; l1–3 – leg pairs 1–3; lg1–4 – leg ganglia 1–4; pa – palp; pg – posterior ganglion; pr – proboscis; sc – scape; seg – subesophageal ganglion; yo—yolk

Postlarval instar 1 is still lecithotrophic as it emerges from the egg membranes and remains attached to the paternal ovigers. It stands out among unhatched embryos, as its body is of ovoid shape (> 300 µm long and ca. 200 µm wide) (Fig. 5A). Its dorsal side is dominated by a copious yolk supply, whereas the ventral side bears the appendages (Fig. 5B–D). A loose cuticle covers the instar (Fig. 5C, D). The cheliphore is directed anteriorly and flanks the short proboscis laterally (Fig. 5C). Externally, its articulation is challenging to discern, but nuclear staining enables to distinguish the proximal scape and distal chela with its two fingers (Fig. 5C, D). The scape bears a short attachment gland spine at its ventrodistal margin. Fibrous secretions emanate from a distal pore on the spine, securing the instar’s attachment to the oviger (Fig. 5C, D). Near the base of the spine, Sytox staining shows a nuclei-free zone, presumably related to the reservoirs of attachment gland cells in the scape (Fig. 5C, D). The palp resembles an elongated, non-articulated bud; a corresponding ovigeral limb bud is missing (Fig. 5C, D). However, a small seta-like cuticular extension overlies the lateral cell region in which the oviger will later on develop (Fig. 5C, D). Similar to postlarval instar 1 of *N. floridanum*, the differentiation of leg pairs 1–3 is externally only incompletely recognizable, as their proximal portions are hidden and coiled under the cuticle (Fig. 5C, D). Due to their compressed condition, the articulation pattern of these leg pairs could not be satisfactorily resolved. The tiny bud of leg pair 4 lies anterolateral to the anlage of the posterior anal tubercle, but is externally hidden under the loose cuticle (Fig. 5C, D). At the posterior tip of the anal tubercle, the proctodeum has started to form (Fig. 5G).

The central nervous system is internally wedged between the anterior and ventral ectoderm and the instar’s copious yolk supply (Fig. 5E). The compact anterior brain anlage is followed by the ventral subesophageal ganglion, four ganglia of the leg-bearing segments and an additional posterior ganglion anlage (Fig. 5E, F). Ventral to leg ganglia 1–3, the corresponding ventral organs resemble compressed cavities, whereas they are externally open invaginations in the less developed leg ganglion 4 and the posterior ganglion (Fig. 5E). Posterior to this, a primordial invagination of another ventral organ marks the beginning formation of a second posterior ganglion (Fig. 5E, F).

#### Postlarval instar 2 (12 × Sytox); Fig. 6

**Fig. 6:**
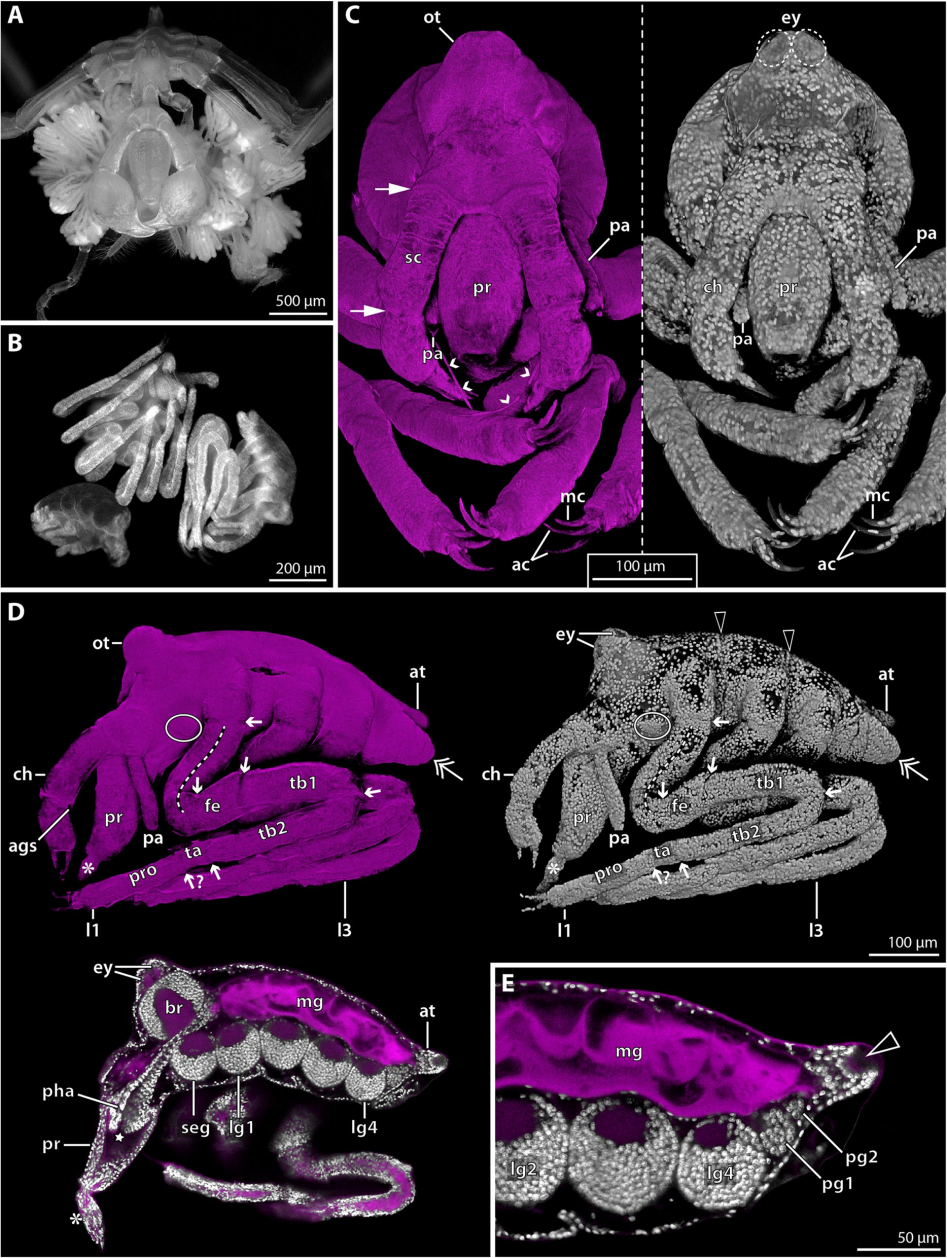
Instar 2 of *Nymphon micronesicum*. **A** Anterior view of adult male bearing > 25 specimens of instar 2; stereomicroscopic autofluorescence image. **B** Comparison of instar 1 (left bottom corner) and two instar 2 specimens after Sytox staining, epifluorescence image. **C-E** CLSM scans of instar 2, autofluorescence (magenta) and Sytox staining (gray). **C** Anterior view (blend mode). The white arrows indicate cheliphoral scape borders. The small arrowheads highlight fibrous secretions from the attachment gland spine. **D** Lateral view (upper row: blend mode; left bottom corner: virtual sagittal section). The oval and the double arrow highlight the buds of oviger and leg 4, respectively. The small arrows indicate podomere borders along leg 1. The stippled line marks coxae 1–3, between which podomere borders could not be confidently identified. The black arrowheads point to trunk segment borders underneath the dorsal cuticular cover. The star and asterisks highlight internal and externally protruding parts of the ruptured pharynx. **E** Magnification of posterior body pole (virtual sagittal section). The arrowhead marks the proctodeum at the tip of the anal tubercle. Abbreviations: ac – auxiliary claw; ags – attachment gland spine; at – anal tubercle; br – brain; ch – cheliphore; ey – eye; fe – femur; l1&3 – leg pairs 1 and 3; lg1–4 – leg ganglia 1–4; mc – main claw; mg – midgut; ot – ocular tubercle; pa – palp; pg1&2 – posterior ganglia 1&2; pha – pharynx; pr – proboscis; pro – propodus; sc - scape; seg – subesophageal ganglion; ta – tarsus; tb1&2 – tibia 1&2

Postlarval instar 2 remains lecithotrophic and attached to the paternal oviger (Fig. 6A). It has reached a body length of more than 400 µm and contrasts starkly to the preceding instar 1 by featuring fully emerged, long leg pairs 1–3 (Fig. 6B). For the first time, the anterodorsal ocular tubercle is observable and the two eye pairs are visible after nuclear staining (Fig. 6C, D). The proboscis is barrel-shaped, with the mouth at its tip (Fig. 6C). In some of the specimens studied, however, a cuticle-covered extension protrudes distally from the tip of a compressed curved proboscis (Fig. 6D). This deviating proboscis shape represents an artifact of the harsh dehydration in ethanol, as internal anatomy reveals it to be caused by the rupture and extrusion of distal pharynx parts (Fig. 6D). The cheliphore is distinctly three-articled and bears the attachment gland spine (Fig. 6D) from which fibrous secretions emanate (Fig. 6C). The palpal limb bud has further elongated, but remains non-articulated (Fig. 6C, D). The oviger primordium can be externally discerned as a minute elevation of the cuticle (Fig. 6D). Corresponding to *N. floridanum*, leg pairs 1 and 2 each bear now the distal main claw and auxiliary claws (Fig. 6C, D), whereas leg pair 3 still lacks these claws. The podomere borders between coxae 1–3 are difficult to discern; the ones of femur, tibia1 and tibia 2 are more reliably identifiable (Fig. 6D). Whether propodus and tarsus are already fully separated could not be resolved. Leg pair 4 resembles a posteriorly directed limb bud containing folded tissue which extends over the anlage of the posterior anal tubercle with the proctodeum at its tip (Fig. 6D, E).

The central nervous system has increased in size, is fully detached from the apical ectoderm and dorsally overlain by the yolk-filled midgut (Fig. 6D, E). The roundish brain and ventral ganglia are distinctly subdivided into a central neuropil core and a surrounding soma cortex (Fig. 6D, E). Two tiny posterior ganglia without distinct neuropil core adjoin leg ganglion 4 posteriorly (Fig. 6E).

## Discussion

### Postlarval hatching stages of Nymphonidae show similarities to Callipallenidae

Prior to this study, data on the development of tropical nymphonids were completely lacking. Our results now reveal that *Nymphon floridanum* and *N. micronesicum* fall into developmental type 5 of postlarval hatchers, as clearly demonstrated by the embryonic formation of (1) the four leg-bearing trunk segments with their ganglia, including the significantly advanced differentiation of leg pairs 1–3, (2) the anlagen of two additional posterior ganglia and (3) the proctodeal invagination at the posterior body pole. Further anatomical correspondences with other postlarval hatchers among Nymphonidae pertain to the structural differentiation of the three anterior head appendages at hatching: the functional cheliphore is followed by a non-articulated palpal limb bud and an inconspicuous oviger primordium [28, 60]. As such, this configuration is not unique to nymphonid postlarval hatchers but closely mirrors a transient condition in advanced free-living instars of anamorphic developers that emerge as protonymphon larva [35, 55, 66]. But in the latter it results from nearly complete atrophy of functional, three-articled palpal and ovigeral larval limbs, and is followed by their de novo outgrowth into the definitive adult palps and ovigers, thus representing re-emerging “Lazarus appendages” [67]. Hence, the distinctive feature of nymphonid postlarval hatchers such as *N. floridanum* and *N. micronesicum* is rather the absence of any articulated palpal and ovigeral larval limbs prior to the non-articulated anlagen present at hatching ([59]; this study). This lack of larval limbs is a developmental feature shared with the advanced hatching stages of Callipallenidae [17, 19, 29, 39], whereas in Pallenopsidae—the only other pycnogonid lineage with members known to display extended embryonic development—they are being formed [27].

Consequently, nymphonid and callipallenid postlarval hatchers are the only sea spiders in which the definitive adult palp (if present) and oviger arise by a unidirectional differentiation process during the development of the palpal and ovigeral segments, comparable to the adult cheliphore (if present) and ambulatory legs.

### Extended embryonic development in sea spiders: convergent cold water adaptation or common evolutionary origin?

Similar to other marine invertebrate taxa [42–44], prolonged brooding of large, lecithotrophic offspring in pycnogonids has been strongly correlated with cold water habitats at higher latitudes or great depth. The only documented examples of lecithotrophic protonymphon-like larvae in the family Ammotheidae stem from several *Ammothea* species from the Southern Ocean [21, 24, 68, 69], as do the only known cases of lecithotrophic postlarval hatchers among Pallenopsidae [27]. In Nymphonidae, all previous reports of lecithotrophic protonymphon-like larvae or postlarval hatchers are likewise confined to Arctic or Antarctic waters [23, 28, 38, 57–60]. The phylogenetic distance between the taxa [3, 14] has led to the assumption that lecithotrophic development emerged multiple times during pycnogonid evolution as a convergent adaptation to low temperature habitats [13, 37, 40].

Until now, Callipallenidae was the only sea spider family known to defy this pattern. Irrespective of latitude, water depth or temperature, callipallenids robustly display prolonged brooding and lecithotrophic hatching stages that resemble advanced postlarval instars rather than a protonymphon larva [13, 19, 29, 39, 70], indicating that their developmental mode is phylogenetically conserved. Our study of *Nymphon floridanum* and *N. micronesicum* now reveals the first two non-callipallenid sea spiders that break the latitudinal trend. Both species are rather small and inhabit shallow tropical waters, challenging the notion that embryonized development and lecithotrophic hatching stages can be solely attributed to cold-water adaptation in Nymphonidae.

In line with pioneering analyses based on single or few gene fragments [71–74], recent molecular phylogenies [3, 14] unanimously support Callipallenidae + Nymphonidae as monophyletic Nymphonoidea (sensu [14, 45]). This may point to a common evolutionary origin of the extended embryonic development not just in Callipallenidae but in both groups, being uniquely characterized by the shared absence of palpal and ovigeral Lazarus appendages (see previous section). Complicating this evolutionary scenario, however, are the suggested interrelationships within Nymphonoidea. Almost all analyses find evidence for Nymphonidae nested within paraphyletic callipallenids [3, 14, 71, 73]. While this suggests that embryonized development evolved at the base of Nymphonoidea, at the same time it enforces the reversion to anamorphic development with a regular larva within Nymphonidae (see [29]).

To satisfactorily resolve the question of independent ecological adaptation versus common ancestry for the extended embryonic development in Nymphonoidea, an improved phylogenetic framework that includes most, if not all nymphonoid genera is key. Attempts to include the two tropical epimorphic developers *N. floridanum* and *N. micronesicum* in state-of-the-art molecular analyses failed so far in the sequencing stage. Future efforts should therefore continue seeking (1) to include a wider range of nymphonid terminals from various latitudes that cover all known types of development, and (2) to expand the coverage of callipallenid diversity, as so far only eight of 17 accepted genera [45] have been analyzed.

### Advantages of fluorescence-based imaging over standard SEM documentation

Building on a previous study on large and robust instars of ethanol-preserved pallenopsids [27], we here exemplarily studied more fragile developmental stages of two nymphonids to assess the potential of autofluorescence imaging and/or fluorescent nuclear staining versus classical SEM for morphological documentation of suboptimally fixed samples. Even under a low-magnification stereomicroscope, the adverse effects of ethanol preservation in the form of artefactual collapse and wrinkling of weakly sclerotized structures (e.g., long appendages) were immediately recognizable (see Fig. 1A). Due to the suboptimal condition of the specimens, reliable description of instar eidonomy by SEM proved highly problematic. Although gradual rehydration in some cases led to an unfolding of the cuticular structures, attempts to subsequently process these samples for SEM invariably resulted in their re-collapse. Similar issues of cuticular collapse and specimen distortion have been reported previously in SEM studies on pycnogonid development, sometimes even after dedicated tissue fixation prior to transfer into ethanol had been performed (e.g. [22, 58, 69]).

Compared to SEM, documentation of cuticular autofluorescence and/or nuclear staining yielded significantly better results in *N. floridanum*, as some of the specimens unfolded during rehydration and remained immersed in aqueous media throughout documentation. Especially in combination with CLSM, this approach yields additional information on internal structures, which are inaccessible to SEM analysis regardless of fixation regime.

Accordingly, when working on rare and fragile pycnogonid instars preserved in high-percentage ethanol (e.g., from bulk samples of research cruises), fluorescence microscopy-based documentation after rehydration appears superior to SEM in terms of reliability and information content of the morphological data obtained and should therefore be given preference (see also [27]). The advantages of autofluorescence imaging have been previously advocated [75] and readily embraced for other arthropod taxa (e.g. [76–79]). But only recently, they have been exploited in first pycnogonid studies [65, 80, 81]. Beyond the preservation-dependent application evaluated here, these studies demonstrate the suitability of this inexpensive, non-invasive method to complement SEM or microCT-based eidonomic documentation of any pycnogonid material, including historical samples held in museum collections.

## Conclusions

The discovery of epimorphic development with lecithotrophic hatching instars in two small *Nymphon* species from tropical shallow waters questions whether this developmental pathway represents an exclusive cold-water adaptation in Nymphonidae. Rather, close phylogenetic affinities of nymphonids and callipallenids may indicate a common evolutionary origin of extended embryonic development in the clade Nymphonoidea. To further test the plausibility of this scenario, the establishment of a stable and well-resolved phylogenetic backbone for Nymphonoidea will prove essential.

Our study demonstrates that non-invasive fluorescence imaging methods yield more reliable morphological data than classical SEM when dealing with delicate developmental stages fixed in ethanol. Accordingly, fluorescence-based approaches are highly recommended for optimizing the documentation of fragile, ethanol-preserved sea spider material, including unsorted bulk samples from research cruises or specimens housed at museum collections.

## Data Availability

The data used and analyzed during the study are included in this published article. Raw datasets are available from the corresponding author upon reasonable request.
